# Supplementary material for: Locus coeruleus noradrenergic neurons phase-lock to prefrontal and hippocampal infra-slow rhythms that synchronize to behavioral events
Source: Front Cell Neurosci. 2023 Mar 21;17:1131151. doi: 10.3389/fncel.2023.1131151 (PMC10070758; doi:10.3389/fncel.2023.1131151)
Supplement: Supplementary file 3 [file Image_3.pdf]

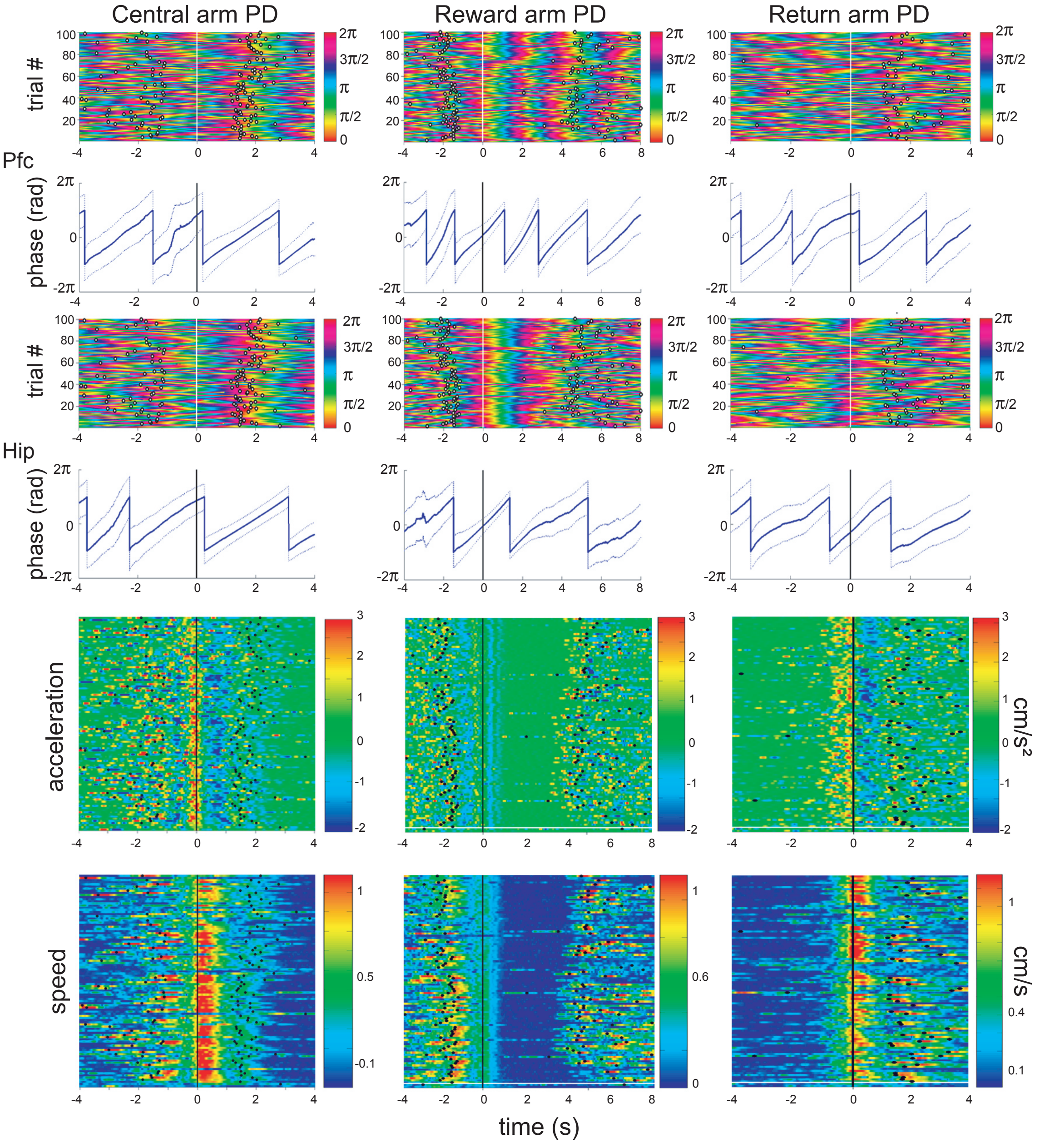

Supplementary Figure 3. Example of lack of a clear relation between speed, acceleration and infra-slow phase. Acceleration increases before central and return arm PD crossings, with speed increasing afterwards. But, the phase is π radians for the former and -0.2*π radians for the latter.
