# Supplementary material for: Locus coeruleus noradrenergic neurons phase-lock to prefrontal and hippocampal infra-slow rhythms that synchronize to behavioral events
Source: Front Cell Neurosci. 2023 Mar 21;17:1131151. doi: 10.3389/fncel.2023.1131151 (PMC10070758; doi:10.3389/fncel.2023.1131151)
Supplement: Supplementary file 5 [file Image_5.pdf]

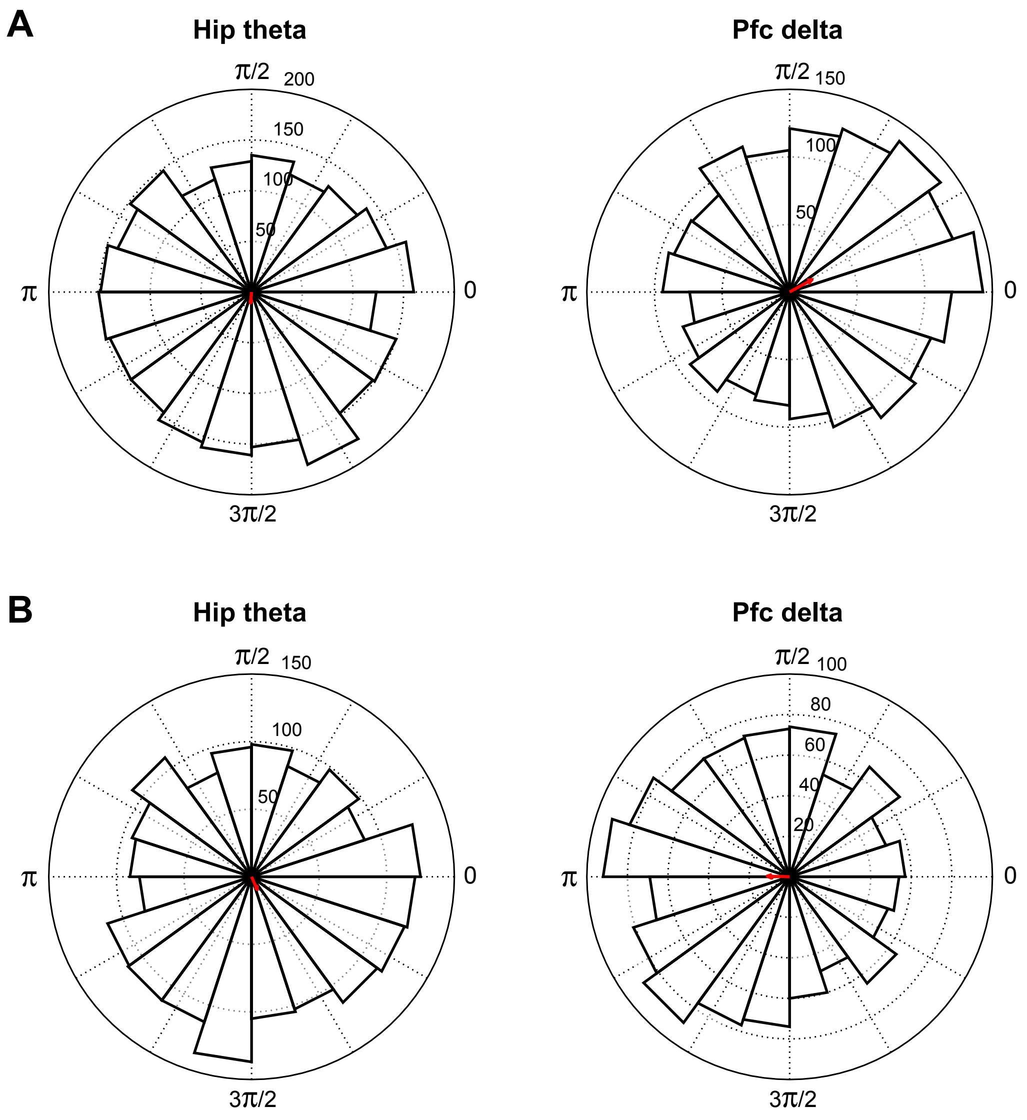

Supplementary Figure 5. Examples of phase-locking of LC neurons to hippocampal theta (filtered 5-10 Hz) and prefrontal delta (filtered 1-4 Hz) rhythms. A and B are two different neurons from two different rats. Same format as Figure 2. A) Hip theta: p=2.6E-4 (Rayleigh test), resultant vector phi=4.6 radians; Pfc delta: p=4.2E-15 (Rayleigh test), phi=0.5 radians, B) Hip theta: p=6.2E-5 (Rayleigh test), phi=5.1 radians; Pfc delta: p=7.4E-8; phi=3.1 radians.
